# Ameliorative Effect of Cactus* (Opuntia ficus indica)* Extract on Lithium-Induced Nephrocardiotoxicity: A Biochemical and Histopathological Study

**DOI:** 10.1155/2017/8215392

**Published:** 2017-12-10

**Authors:** Anouar ben Saad, Ilhem Rjeibi, Sana Ncib, Nacim Zouari, Lazhar Zourgui

**Affiliations:** ^1^Research Unit of Macromolecular Biochemistry and Genetics, Faculty of Sciences of Gafsa, University of Gafsa, 2112 Gafsa, Tunisia; ^2^Research Unit of Active Biomolecules Valorisation, Higher Institute of Applied Biology of Medenine (ISBAM), University of Gabes, 4119 Medenine, Tunisia; ^3^Unit of Common Services, Faculty of Sciences of Gafsa, University of Gafsa, 2112 Gafsa, Tunisia; ^4^Higher Institute of Applied Biology of Medenine (ISBAM), University of Gabes, 4119 Medenine, Tunisia

## Abstract

*Opuntia ficus indica* (family Cactaceae) is used in the treatment of a variety of conditions including metal-induced toxicity. The study reports the protective effects of* Opuntia ficus indica* (CCE) against lithium carbonate-induced toxicity in rats. Nephrocardiotoxicity was induced in male Wistar rats by single dose of lithium carbonate (25 mg/kg b.w twice daily for 30 days). Aqueous extract of* Opuntia ficus indica* was administered at the dose of 100 mg/kg of b.w by gavage for 60 days. Obtained results revealed that administration of lithium carbonate caused a significant increase in serum creatinine, uric acid, and urea levels. Additionally, a significant decrease in the level of renal and cardiac SOD, CAT, and GPx activities was associated with a significant increase of MDA levels in lithium carbonate group more than those of the control. However, the treatment of experimental rats with CCE prevented these alterations and maintained the antioxidant status. The histopathological observations supported the biochemical evidences of nephrocardioprotection. CCE supplementation could protect against lithium carbonate-induced renal and cardiac injuries in rats, plausibly by the upregulation of antioxidant enzymes and inhibition of MDA to confer the protective effect.

## 1. Introduction

Bipolar disorder is a manic depressive illness and is characterized by alternative periods of elevated mood and depression. It is one of the leading causes of mental disability in the world. Lithium salts have been widely used as mood stabilizers for the treatment of bipolar disorders and depression [1]. Long term lithium therapy is mostly associated with many side effects affecting liver, heart, testes, and kidney functions [2]. Lithium salts cause ocular side effects: polydipsia, polyuria, and diabetes insipidus. Oxidative stress has been proposed as an alternative mechanism of lithium toxicity in certain animal tissues, via impairment of mitochondrial respiratory system that leads to increased generation of free radicals through lipid peroxidation of cell membrane [3–6]. Since free radical generation is expected to induce organ toxicities including nephrocardiotoxicity, supplementation with antioxidants may improve tissue capacity to cope with the high antioxidant demands [7, 8].

Therefore, safe and effective natural products that may confer free radical scavenging activities are in global demand as an additional armamentarium against oxidative damage [8, 9].

The genus* Opuntia* belongs to the Cactaceae family, comprising about 200 species all over the world [10]. Plants from the* Juniperus* genus have found application in different European cuisines, as well as in cosmetics [10]. Furthermore, these plants have an extensive history of use in global folk medicine for numerous chronic diseases, including cancer as well as cardio- and cerebrovascular, ocular, and neurological diseases [11, 12]. Among their species,* Opuntia ficus indica* is widely distributed in Latin America, South Africa, and the Mediterranean area. It can be used as anti-inflammatory, analgesic, hypoglycemic, antiviral, and antioxidant [13–15]. Previous phytochemical studies reveal that* Opuntia ficus indica* contains a large variety of compounds, mainly vitamins, carotenoids, fatty acids, and essential oil [16, 17].

To our knowledge, there are no data about the* in vivo* effect of cactus* O. ficus indica* cladodes extract on male renal and cardiac damage and oxidative stress induced by lithium carbonate. The present study aimed to investigate whether a diet enriched with* O. ficus indica* cladodes extract could inhibit lithium carbonate-induced renal and cardiac damage and oxidative stress using Wistar rats as animal model.

## 2. Material and Methods

### 2.1. Chemicals

All the chemicals are of analytical grade and were purchased from E-Merck (Germany), Sigma Aldrich, Scharlau, NORMAPUR, BIOTECH, Panreac, CHIMICA PLUS, and so forth.

### 2.2. Preparation of* Opuntia ficus indica* Extract

Young cactus cladodes of* Opuntia ficus indica* (2-3 weeks old) were collected at the beginning of March 2014 in Gafsa, state of Tunisia. A voucher specimen (OFI 0214) was identified and authenticated by a taxonomist and deposited at the herbarium (H03) in the Faculty of Sciences, University of Gafsa, Tunisia. The sample was washed with water, cut into small pieces, then pressed using a hand press, homogenized with 10 Mm Tris-HCl, pH 7.4, at 4°C, and centrifuged for 30 min at 3500*g* at 4°C. The supernatant was subsequently collected and lyophilized. The CCE yielded 22.82*g* of green residue and was stored at −21°C until use. The yielded CCE is totally soluble when dissolved in distilled water and gives a slightly viscous green solution.

### 2.3. Animals and Treatments

Two-month-old healthy male Wistar rats (*n* = 24) weighing about 120 ± 10 g were purchased from Central Pharmacy of Tunis (Tunisia) and maintained for an adaptation period of 1 week under the same conditions of temperature (22 ± 2°C), relative humidity (70 ± 4%), and 12 h light/dark cycle. The animals were fed commercial pellet diet and tap water ad libitum. The handling of the animals was approved by the Medical Ethics Committee for the Care and Use of Laboratory Animals of the Pasteur Institute of Tunis (approval number: FST/LNFP/Pro 152012) and carried out according to the European convention for the protection of living animals used in scientific investigations (Council of European Communities 1985). After the adaptation period, animals were divided into four groups of six rats each and treated as follows:

group (C): control rats given distilled water (0.5 ml/100 g of body weight; i.p.);

group (Li): rats administered intraperitoneally (i.p.) with 25 mg/kg of lithium carbonate (dissolved in distilled water) twice daily for 30 days [18];

group (CCE): rats given CCE at 100 mg/kg of b.w. for 60 days and then injected with distilled water (0.5 ml/100 g b.w.; i.p.) during the last 30 days of CCE treatment;

group (Li + CCE): rats given CCE at 100 mg/kg of b.w. for 60 days and then injected with lithium carbonate at a dose 25 mg/kg of b.w. (i.p.) during the last 30 days of CCE treatment.

### 2.4. Serum Sample Collection

After 60 days of treatment, the control and treated groups were sacrificed by decapitation under ether inhalation anesthesia in order to minimize the handling stress. The blood serum was obtained by centrifugation (1,500 rpm, 15 min, 4°C) and stored at −80°C until use for biochemical determination.

### 2.5. Tissue Preparation

During the treatment period, the body weight of the animals was monitored daily. On the day of sacrifice, kidney and heart of rats were quickly removed, cleaned, weighted, and used for biochemical and histological studies. Then relative weight organ was calculated. Kidney and heart were used for histological analyses, lipid peroxidation, and antioxidant analyses. Oxidative status in kidney and heart could be estimated from the concentrations of lipid peroxidation (LPO), total sulfhydryl groups (TSH), and the activities of three representative antioxidant enzymes: superoxide dismutase (SOD), catalase (CAT), and glutathione peroxidase (GPx). To measure these indicators, renal and cardiac tissues were cut into small pieces and immersed into a 2-ml ice-cold lysis buffer (TBS, pH 7.4); the mixtures were homogenized on ice using an Ultra-Turrax homogenizer for 15 min and then filtered and centrifuged (5,000 rpm, 30 min, 4°C). Supernatants (S1) were collected and stored at −80°C until use.

### 2.6. Biochemical Assays

#### 2.6.1. Assays of Serum Markers

The levels of creatinine, uric acid, and urea in serum were determined by kit methods (Spinreact, Girona, Spain).

#### 2.6.2. Analysis of the Level of Lipid Peroxidation

The lipid peroxidation level in liver was measured as the amount of thiobarbituric acid reactive substance (TBARS) according to the method of Ohkawa et al. [19]; 125 *μ*l of supernatants (S1) was homogenized by sonication with 50 *μ*l of TBS and 125 *μ*l of TCA-BHT to precipitate proteins and centrifuged (1000 rpm, 10 min, and 4°C); 200 *μ*l of supernatant was mixed with 40 *μ*l of HCl (0.6 M) and 160 *μ*l of TBA dissolved in Tris, and the mixture was heated at 80°C for 10 min. MDA forms adducts with TBA, which is measured spectrophotometrically at 532 nm. MDA, a product of LPO, was used as a standard. The amount of MDA was calculated by using an extinction coefficient of 156 Mm^−1 ^cm^−1^ and expressed in nmoles/mg protein.

#### 2.6.3. Total Sulfhydryl Groups (TSH)

Tissue TSH were measured spectrophotometrically at 412 nm in liver homogenates after reaction with 5,5%-dithiobis-(2-nitrobenzoic acid). Thiol content was expressed in U/mg protein [20].

#### 2.6.4. The Activities of Antioxidant Enzymes

Superoxide dismutase (SOD) activity was determined by measuring its ability to inhibit the photoreduction of NBT [21]. Catalase (CAT) activity was assayed spectrophotometrically as described by Aebi [22]; the H_2_O_2_ decomposition rate was followed by monitoring absorption at 240 nm. The GPx activity was evaluated according to the method of Flohe and Gunzler [23]. The activity of GPx was expressed in *µ*mol of GSH oxidized/min/g of protein, at 25°C. The content of proteins in the tissue extracts was measured by the method of Lowry et al. [24] using the bovine serum albumin (BSA) as standard.

#### 2.6.5. Histopathological Analysis

Parts of kidney and heart were quickly excised and immersed for 48 h at 4°C in a fixative solution (10% formaldehyde, in phosphate buffer, pH 7.6), dehydrated in ethanol, and embedded in paraffin. Paraffin sections (5–8 *μ*m thick) were made and stained with hematoxylin-eosin solutions (H&E). Tissues preparations were observed and microphotographed under a light BH2 Olympus microscope (Olympus, Tokyo, Japan).

### 2.7. Statistical Analysis

All data were presented as means ± standard deviation (SD). Determination of all parameters was performed from six animals per group. Significant differences between treatment effects were determined using one-way ANOVA, followed by Tukey's HSD post hoc tests for multiple comparisons with statistical significance of *p* < 0.05.

## 3. Results

### 3.1. Body Weight

**Figure 1:**
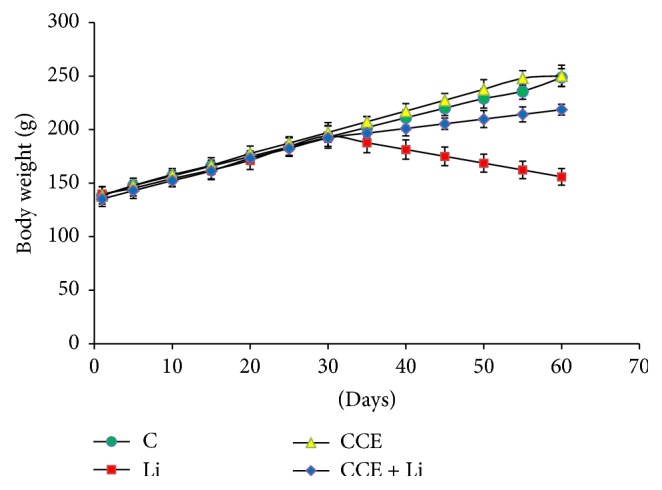
Effects of lithium on body weight of male rats. Values were expressed as mean ± SEM of 6 rats per group: controls or treated with lithium (Li), cactus cladodes extract (CCE), cactus cladodes extract + lithium (Li + CCE) during 60 days.

**Figure 2:**
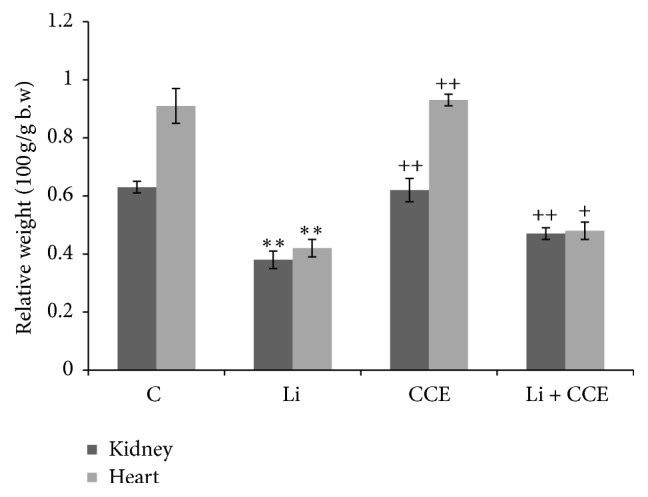
The effects of CCE and lithium carbonate on BW and relative organ weights. Values were expressed as mean ± SEM of 6 rats in group. ^*∗∗*^*p* < 0.01 compared with control (C). ^+^*p* < 0.05. ^++^*p* < 0.01 compared with lithium carbonate-treated group (Li).

No death was observed in any of the experimental groups. Figures 1 and 2 show the effects of CCE and lithium carbonate on the body and organ weights in different experimental groups. The gain in body weight was less in lithium-treated rats (155.9 ± 6.32 g) than the control animals (248.7 ± 10.20 g). However, pretreatment with CCE in combination with lithium carbonate increased the body weight gain in rats (213.1 ± 5.90 g) compared with lithium-carbonate treated group. Concerning relative kidney and heart weight, hypotrophies of these organs were observed when animals were intoxicated with lithium carbonate. Yet, this is not noticed neither when CCE is joined to lithium carbonate nor when it was administrated alone.

### 3.2. Assays of Serum Markers

As shown in Table 3, lithium treatment (25 mg/kg b.w) induced a significant increase (*p* < 0.01) of creatinine and urea and significant decrease of uric acid as compared to controls. When lithium-treated rats were also treated with CCE, all these biomarkers were kept to almost normal values.

### 3.3. Lipid Peroxidation

Based on the investigation of oxidative stress biomarkers, there was a significant increase in LPO levels in the animals treated with lithium alone, as evidenced by the increase in kidney and heart MDA levels compared to the control group. However, CCE conferred a protective effect because animals supplemented with CCE extract had significantly (*p* < 0.01) lower MDA levels when compared with lithium carbonate-treated group. The extract alone did not show significant effect on LPO (Table 4).

### 3.4. Antioxidative Activities

TSH levels of control and experimental rats are shown in Table 4. A significant decrease (*p* < 0.01) in the level of this nonenzymatic antioxidant was noticed in rats treated only with lithium carbonate when compared with normal rats. Pretreatment with CCE significantly (*p* < 0.01) restored the levels of TSH to near normal when compared with lithium carbonate-treated animals.

The effects of administration of lithium carbonate for 30 days on antioxidant enzyme activities in kidney and heart tissues are shown in Table 4. The enzyme, such as SOD, CAT, and GPx, activities were significantly (*p* < 0.01) decreased in animals which received lithium carbonate only when compared with normal controls. However, administration with CCE to lithium carbonate-treated rats significantly ameliorated the levels of SOD, CAT, and GPx enzymes when compared with rats treated with lithium carbonate.

### 3.5. Histopathological Examination

**Figure 3:**
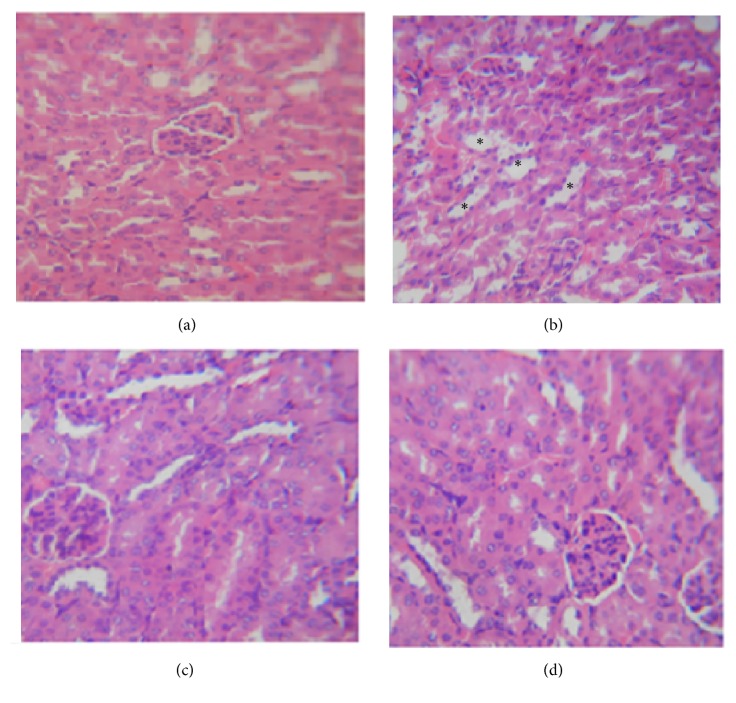
Light micrographs of hematoxylin-eosin (H&E) stained kidney paraffin sections (×400): (a) control group showing normal histological structures; (b) lithium-treated group showing necrosis of renal cells (*∗*), severe degenerative changes in tubules, and damaged glomeruli; (c) the normal kidney structure of rats treated with CCE; (d)* O. ficus indica* cladodes extract with lithium group showing marked improvement in the histological picture with minimal tubules damage and normal structure of the glomeruli.

The kidney section in normal control animals indicated the presence of normal morphology of renal parenchyma, glomeruli, and tubules (Figure 3(a)). In contrast, the lithium-treated rats showed kidney sections with severe damage characterized by necrosis of renal cells, severe degenerative changes in tubules, and damaged glomeruli (Figure 3(b)). However, the kidney sections of animals administered CCE at the dose of 100 mg/kg (b.w) showed moderate degree of kidney damage and normal glomeruli, presence of mild degenerating tubules, and regeneration of renal cells in marked improvement of the tubular damage (Figure 3(d)), whereas the histopathological examination of the kidney CCE-treated group showed normal morphology of glomeruli and tubules comparable to the normal control group (Figure 3(c)).

**Figure 4:**
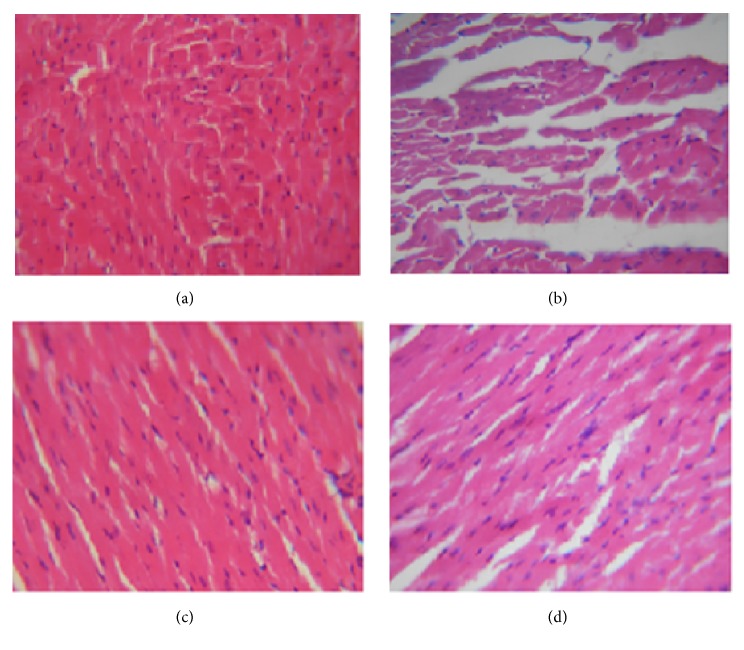
Sections of heart tissue obtained from rats of control groups and treated with CCE in lithium carbonate-induced rats (H and E, ×400). Heart tissue of the control rats showed normal myocardial fibers and muscle bundles with normal architecture (a). Heart tissue of lithium-treated rats showed separation of myocardial fibers with inflammatory mononuclear collections, edema, and myocardial necrosis (b). Myocardial section of CCE-treated rats showed slightly separated myocardial fibers with small focus of inflammatory mononuclear collections with the absence of necrotic damage (d). CCE alone-treated rats showed normal myocardial fibers with no pathological changes (c).

Heart tissue of the control rats showed normal myocardial fibers and muscle bundles with normal architecture (Figure 4(a)). Heart tissue of lithium-treated rats showed separation of myocardial fibers with inflammatory mononuclear collections, edema, and myocardial necrosis (Figure 4(b)). Myocardial section of CCE-treated rats showed slightly separated myocardial fibers with small focus of inflammatory mononuclear collections with the absence of necrotic damage (Figure 4(d)). CCE alone-treated rats showed normal myocardial fibers with no pathological changes (Figure 4(c)).

## 4. Discussion

In our previous study [4] we have shown that* Opuntia ficus indica* extract is rich in natural antioxidants compounds and exhibits an excellent antioxidant activity (Tables 1 and 2). Moreover, we have shown that HPLC analysis of CCE revealed the presence of phenolic acids and flavonoids. There were six phenolic acids identified in CCE which include gallic acid, catechin, caffeic acid, epicatechin, vanillic acid, and coumarin acid with percentage of compounds 10.01%, 7.10%, 3.80%, 6.30%, 11.20%, and 6.50%, respectively, and revealed four flavonoids absorbed at 360 nm including rutin (9.80%), isorhamnetin (4.20%), quercetin (15.02%), and kaempferol (7.92%).

Recently* in vitro* nephroprotective and cardioprotective activity of plant extract has gained much importance. For primary screening, lithium carbonate is typically used to induce* in vivo *kidney and heart toxicity, followed by testing of plant based drugs for their kidney and heart protecting activity.

In the present study, change in rats body weight along with relative organ weights provided an imperative indication of lithium-induced toxicity. Lithium treatment for 30 days caused slight decrease in rats body weight which might be due to the direct cytotoxic effects of the metal on somatic cells and indirectly acting via the central nervous system which controls feed and water intake and regulates the endocrine system, all of which result in decreased appetite and reduced absorption of nutrients from the gut [25–27]. Moreover, the decrease in the relative kidney and heart weights following lithium exposure as observed in our study could be attributed to the relationship between the kidney and heart weight increase and toxicological effects such as edema or to the reduced body weight gain in the experimental animals [3, 28]. However, pretreatment of CCE given to lithium carbonate-treated animals ameliorated the body and kidney and heart weights, which could be attributed either to the phytochemical content in CCE or its antioxidant properties.

Result showed also that lithium-induced nephrotoxicity was characterized by the change in the levels of creatinine, urea, and uric acid. Indeed, high serum creatinine, urea, and uric acid levels can be due to kidney damage [18, 28]. Pretreatment of the rats with CCE significantly ameliorated creatinine, urea, and uric acid levels which can be attributed to the presence of high amount of phenolics and flavonoids [29]. LPO is a well-established mechanism for oxidative stress, leading to cell injury. MDA is one of the most widely used indicators of the cellular redox state [30] which was observed to be elevated in the rat's kidney and heart receiving lithium carbonate. Increased LPO appears to be the initial stage where the kidney and heart tissues become susceptible to oxidative damage. Indeed, in our previous work we have shown that CCE exhibited an excellent* in vitro* antioxidant capacity [31]. Results of the present work showed that the administration with CCE significantly reduced the levels of lipid peroxidation in lithium carbonate-exposed rats suggesting its protective effects against oxidative damage, which confirm its antioxidant capacity. Antioxidants constitute the foremost defense system that limits the toxicity associated with free radicals. The organ had enzymatic and nonenzymatic systems to neutralize free radicals. The nonenzymatic antioxidants such as TSH, ascorbic acid, and reduced glutathione as well as enzymes like SOD, CAT, and GPx are the main antioxidants [32], limiting the effects of oxidant molecules in tissues and acting in the defense against oxidative cell injury by means of their being free radical scavengers [33]. In the present study, lithium carbonate was found to decrease renal and cardiac TSH levels and antioxidant enzymes activities (SOD, CAT, and GPx). As these enzymes had a protective role against oxygen free radical induced damage, their induction could be understood as an adaptative response to oxidative stress [34]. Supplementation of CCE in the diet of lithium carbonate-treated rats could protect the alteration. In fact, it increased the concentration of TSH and thus increased intracellular antioxidant power. We have also observed the restoration of the activities of antioxidants enzymes in lithium carbonate rats pretreated with CCE. Such results are in a good accordance with those obtained by Hfaiedh et al. [35] and Smida et al. [36]. On the other hand, the renal histoarchitecture of the lithium carbonate-treated rats resulted in severe necrotic changes, degenerative changes in tubules, and damaged glomeruli. The administration of CCE reducing the histological alterations provoked by lithium carbonate was quit noticeable. The histological changes seen in the heart of rats treated with lithium carbonate were characterized by a separation of myocardial fibers with inflammatory mononuclear collections, edema, and myocardial necrosis. Our results confirmed previous finding of Abdellatief et al. [37] and Daoud et al. [38] who had found degenerative changes in heart of rat exposed to isoproterenol and cisplatin. The results suggest that CCE treatment prior to lithium carbonate intoxication could prevent the lithium carbonate-induced alterations in heart tissues of treated animals.

## 5. Conclusion

Thus, the finding of this study showed that the administration of the* O. ficus indica* extract appeared to protect the kidneys and heart due to presence of high antioxidant phytochemicals and properties of male rats from lithium carbonate-induced oxidative stress by reducing the intensity of LPO and by enhancing the activities of enzymatic and nonenzymatic antioxidants. In future, work will be done to isolate bioactive constituents of* O. ficus indica* extract to locate potential pharmacological agents.

## Figures and Tables

**Table 1:** Main detected compounds by HPLC from cactus cladode extract (Saad et al. [4]).

Compounds	%
Gallic acid	10.01
Catechin	7.10
Caffeic acid	3.80
Epicatechin	6.30
Vanillic acid	11.20
Coumaric acid	6.50
Rutin	9.80
Isorhamnetin	4.20
Quercetin	15.02
Kaempferol	7.92

**Table 2:** Phenolic compounds and antioxidant activities of cactus cladode extract (Ben Saad et al. [4]).

	TPC^a^ (mg GAE/g CCE)	TFC^b^ (mg QE/g CCE)	DPPH^c^ (mg/ml)	IC50 reducing power (mg/ml)	IC50 chelating activity (mg/ml)
CCE	125.01 ± 0.90	71.02 ± 0.757	0.30 ± 0.03	0.36 ± 0.08	0.49 ± 0.03

^a^Total phenolic content as gallic acid equivalent. ^b^Total flavonoid content as quercetin equivalent. ^c^DPPH radical scavenging activity.

**Table 3:** Effect of lithium and cactus cladode extract on creatinine (U/l), uric acid (U/l), and urea (U/l) levels in blood.

	C	Li	CCE	CCE + Li
Creatinine	20.03 ± 1.56	50.20 ± 1.23^*∗∗*^	21.03 ± 0.9^++^	44.12 ± 1.1^+^
Uric acid	101 ± 0.9	70.3 ± 0.5^*∗∗*^	110.3 ± 1.2^++^	93 ± 1.03^++^
Urea	4.77 ± 0.66	9.12 ± 1.36^*∗∗*^	4.23 ± 0.9^++^	6.02 ± 1.01^++^

Values were expressed as mean ± SEM of 6 rats in group; ^*∗∗*^*p* < 0.01 compared with control (C); ^+^*p* < 0.01, ^++^*p* < 0.01 compared with lithium carbonate-treated group (Li).

**Table 4:** Kidney and heart malondialdehyde levels, nonenzymatic antioxidant levels (TSH), and enzymatic antioxidant activities (glutathione peroxidase, catalase, and superoxide dismutase) in male rat controls or those treated with lithium (Li), cactus cladodes extract (CCE), and cactus cladodes extract + lithium (CCE + Li) during 60 days.

Parameters	C	Li	CCE	CCE + Li
MDA (nmol/mg protein)				
Heart	0.58 ± 0.28	1.55 ± 0.20^*∗∗*^	0.54 ± 0.29^++^	1.03 ± 0.08^++^
Kidney	2.3 ± 0.12	6.12 ± 0.26^*∗∗*^	2.35 ± 0.3^++^	3.4 ± 0.24^++^
SOD (U/mg of protein)				
Heart	9 ± 0.12^++^	4.01 ± 0.21^*∗∗*^	9.59 ± 0.42^++^	5.78 ± 0.15^+^
Kidney	7.23 ± 0.8	3.01 ± 0.12^*∗∗*^	8.56 ± 0.56^++^	6.9 ± 0.9^++^
CAT (*μ*mol/min/mg of protein)				
Heart	15.22 ± 3.21	9.12 ± 2.34^*∗∗*^	15.89 ± 3.6^++^	14.03 ± 4.2^++^
Kidney	13,2 ± 1.7	8,02 ± 0.5^*∗∗*^	14.03 ± 0.45^++^	9.98 ± 0.52^+^
GPx (*μ*mol GSH oxidized/min/mg of protein)				
Heart	6.27 ± 0.28	3.61 ± 0.19^*∗∗*^	5.90 ± 0.53^++^	5.03 ± 0.30^++^
Kidney	3,2 ± 0.12	1,15 ± 0.10^*∗∗*^	4.02 ± 0.9^++^	2.90 ± 0.6^+^
TSH (U/mg protein)				
Heart	1.8 ± 0.09	0.06 ± 0.1^*∗∗*^	1.9 ± 0.08^++^	0.09 ± 0.06^++^
Kidney	1.52 ± 0.1	0.53 ± 0.02^*∗∗*^	1.65 ± 0.03^++^	0.9 ± 0.12^++^

Values were expressed as mean ± SEM of 6 rats in group; ^*∗∗*^*p* < 0.01 compared with control (C); ^+^*p* < 0.01, ^++^*p* < 0.01 compared with lithium carbonate-treated group (Li).
